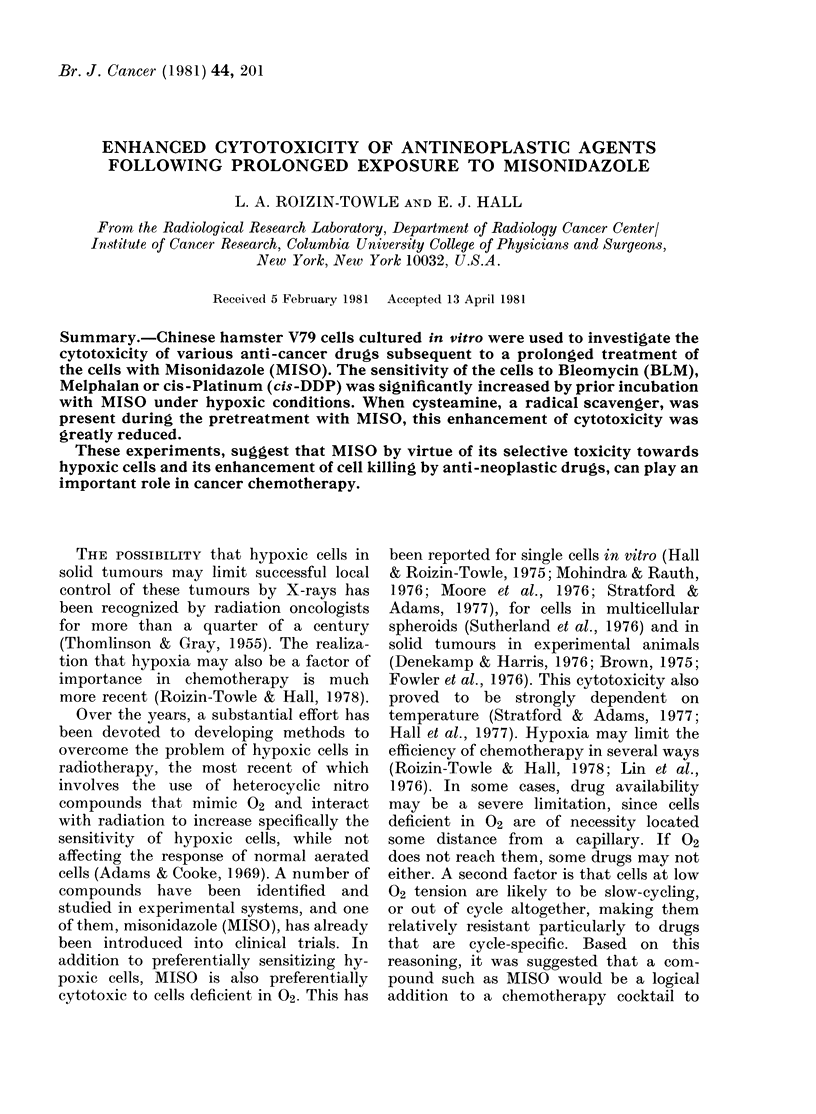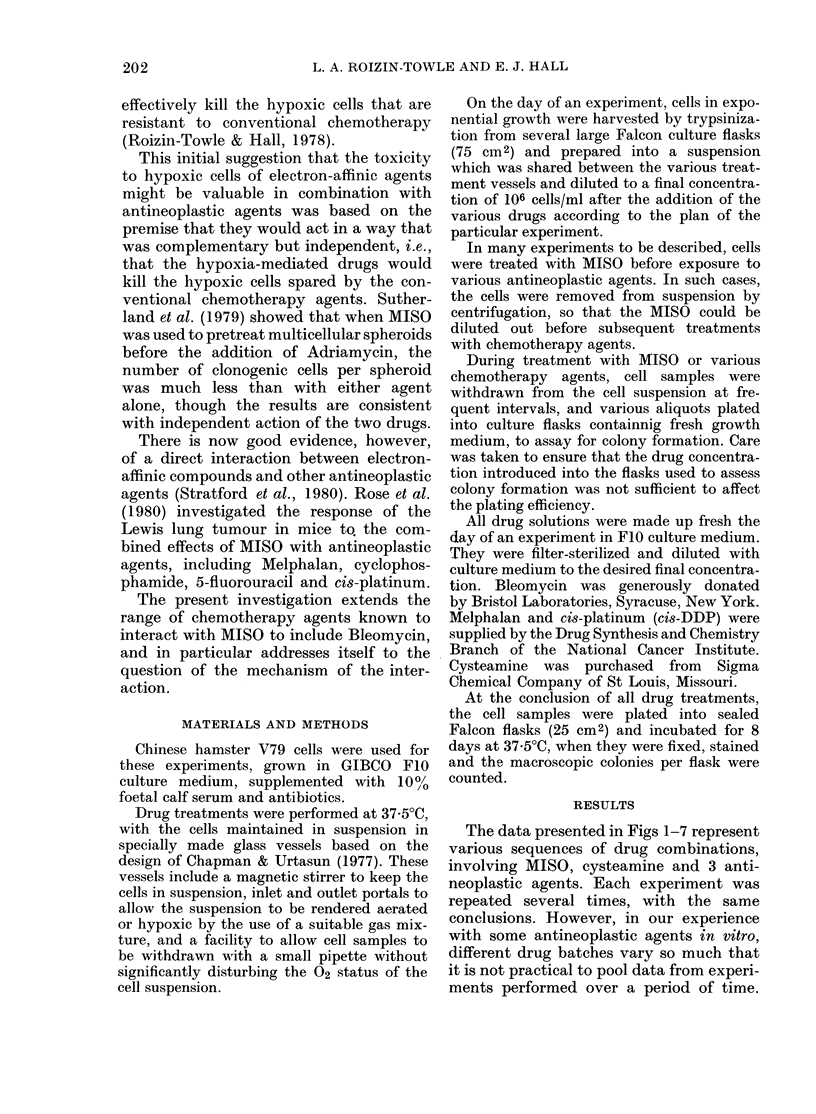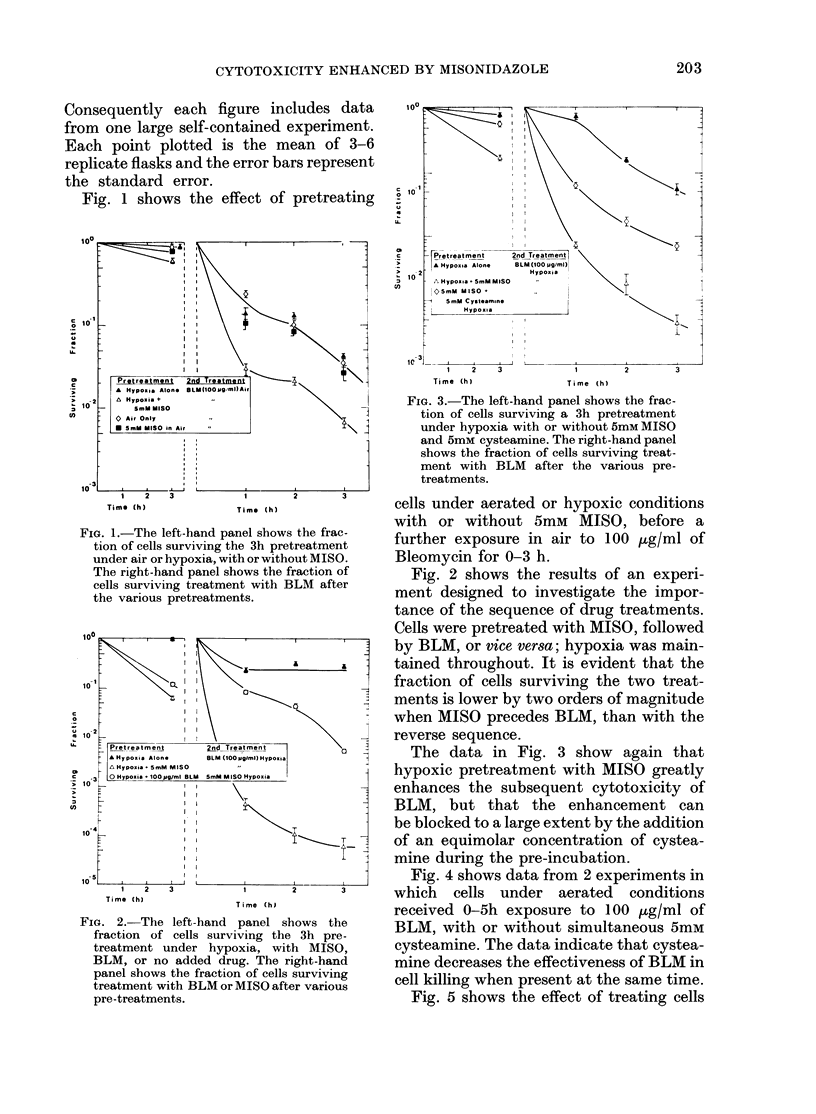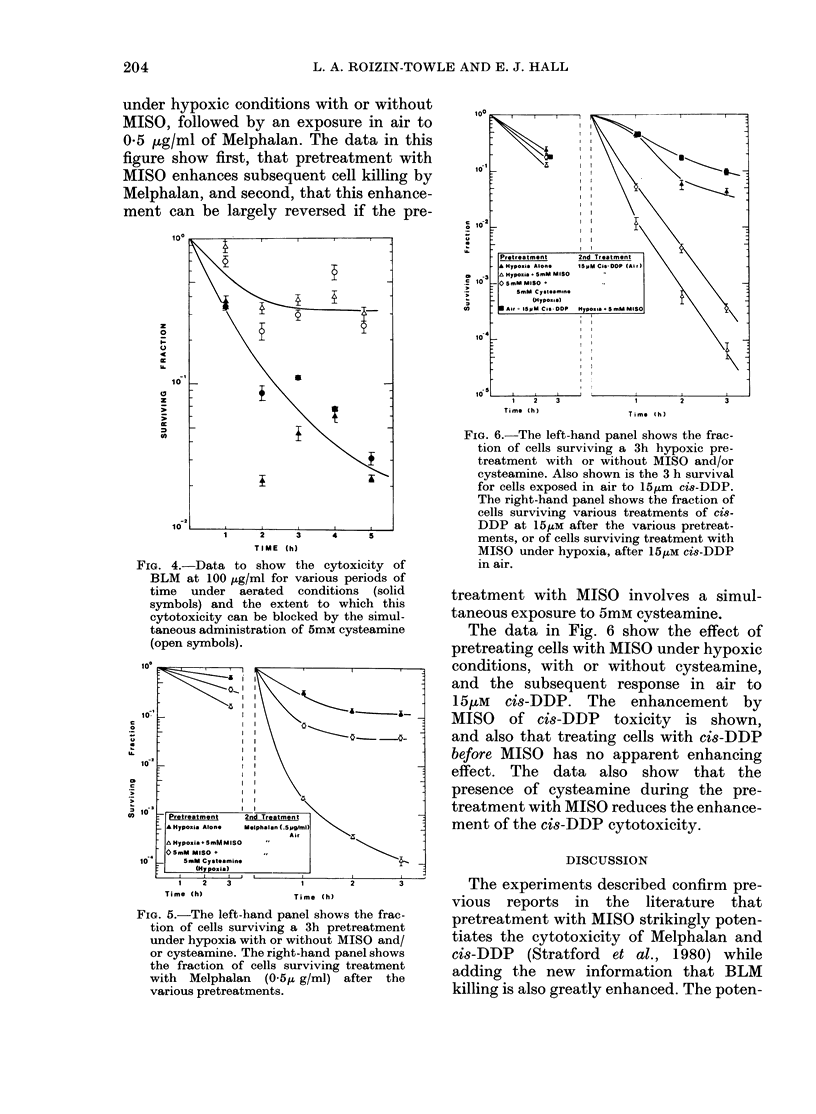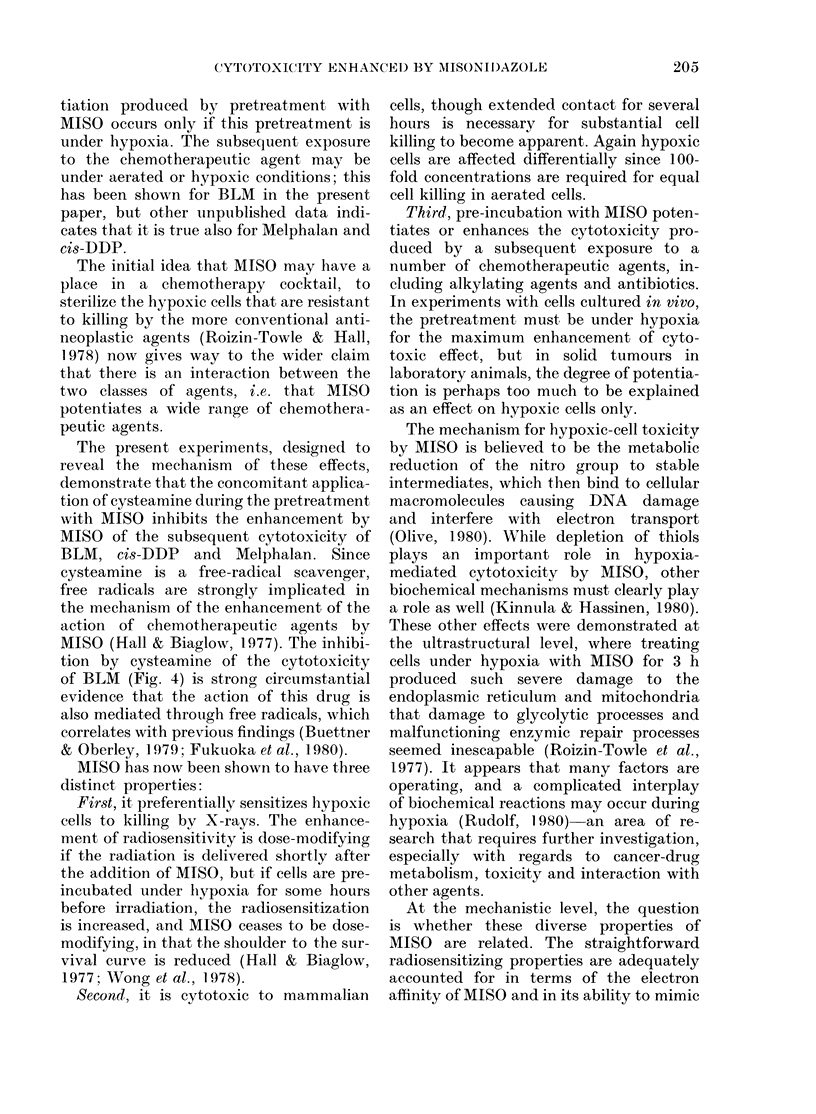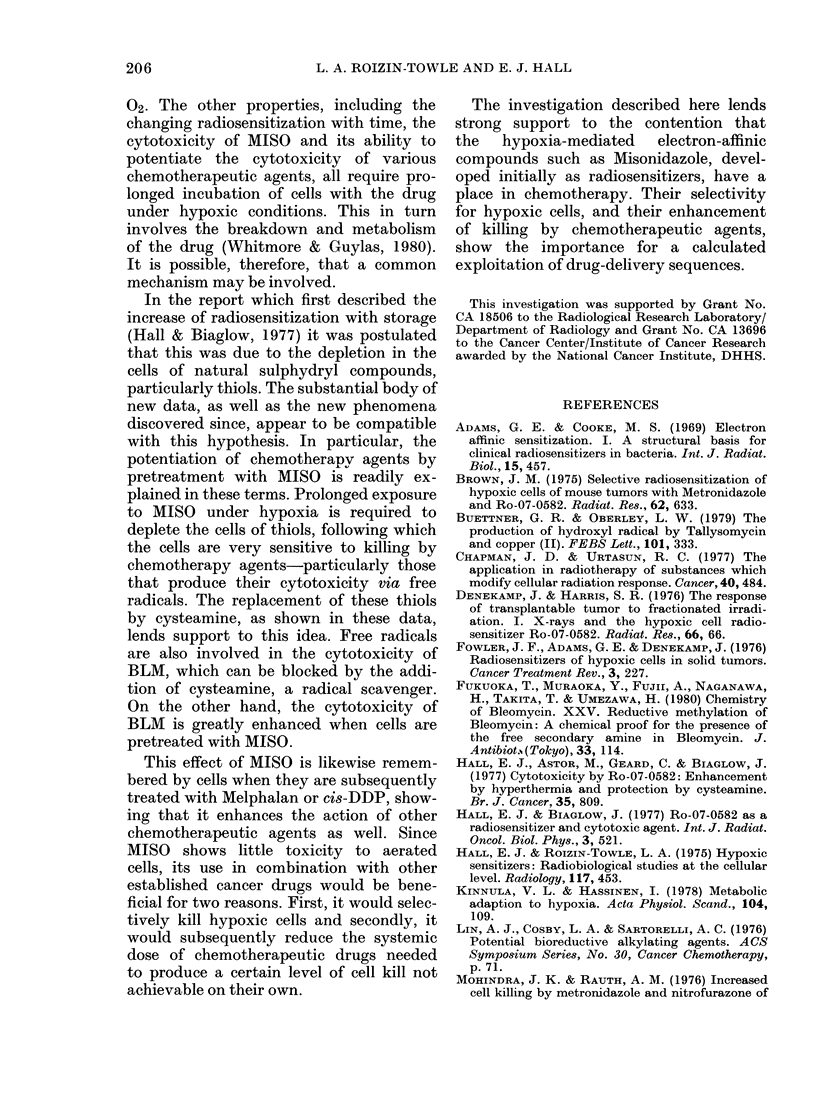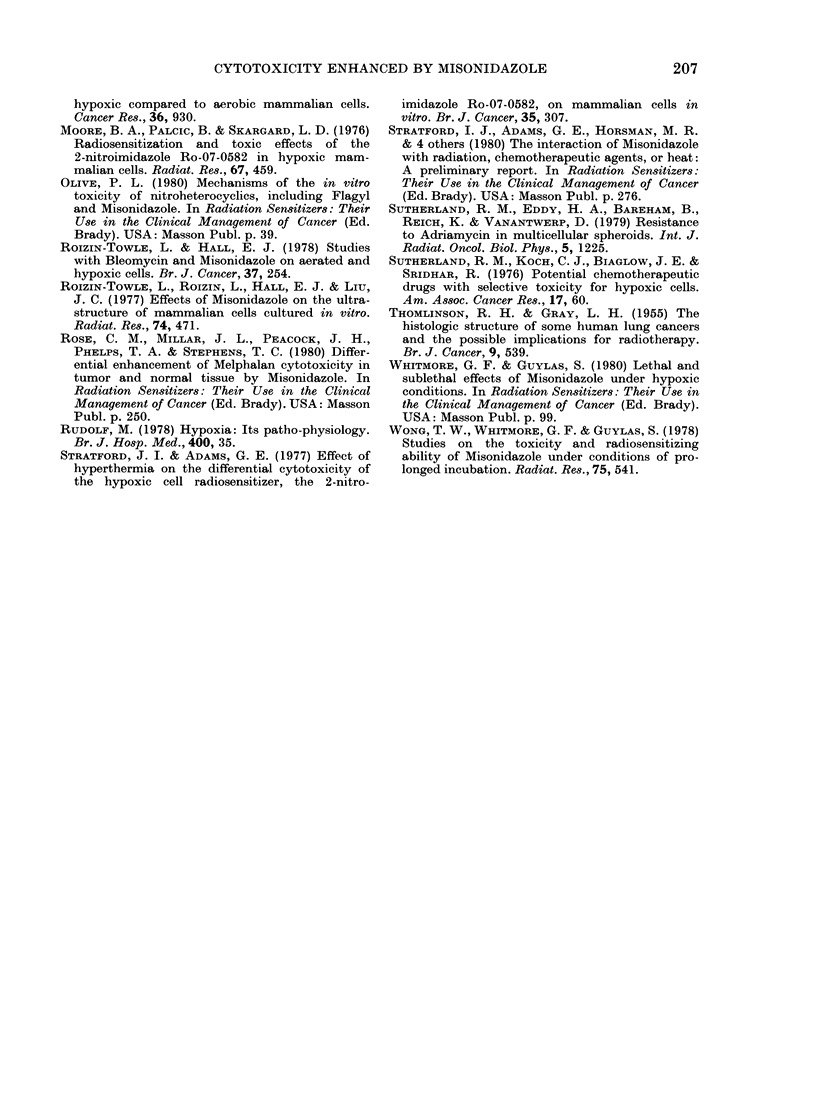# Enhanced cytotoxicity of antineoplastic agents following prolonged exposure to misonidazole.

**DOI:** 10.1038/bjc.1981.171

**Published:** 1981-08

**Authors:** L. A. Roizin-Towle, E. J. Hall

## Abstract

Chinese hamster V79 cells cultured in vitro were used to investigated the cytotoxicity of various anti-cancer drugs subsequent to a prolonged treatment of the cells with Misonidazole (MISO). The sensitivity of the cells to Bleomycin (BLM), Melphalan or cis-Platinum (cis-DDP) was significantly increased by prior incubation with MISO under hypoxic conditions. When cysteamine, a radical scavenger, was present during the pretreatment with MISO, this enhancement of cytotoxicity was greatly reduced. These experiments, suggest that MISO by virtue of its selective toxicity towards hypoxic cells and its enhancement of cell killing by anti-neoplastic drugs, can play an important role in cancer chemotherapy.


Br. J. Cancer (1981) 44, 201

### ENHANCED CYTOTOXICITY OF ANTINEOPLASTIC AGENTS FOLLOWING PROLONGED EXPOSURE TO MISONIDAZOLE

L. A. ROIZIN-TOWLE AND E. J. HALL

*From the Radiological Research Laboratory, Department of Radiology Cancer Center/ Institute of Cancer Research, Columbia University College of Physicians and Surgeons, New York, New York 10032, U.S.A.*

Received 5 February 1981 Accepted 13 April 1981

**Summary.—Chinese hamster V79 cells cultured *in vitro* were used to investigate the cytotoxicity of various anti-cancer drugs subsequent to a prolonged treatment of the cells with Misonidazole (MISO). The sensitivity of the cells to Bleomycin (BLM), Melphalan or cis-Platinum (*cis*-DDP) was significantly increased by prior incubation with MISO under hypoxic conditions. When cysteamine, a radical scavenger, was present during the pretreatment with MISO, this enhancement of cytotoxicity was greatly reduced.**

**These experiments, suggest that MISO by virtue of its selective toxicity towards hypoxic cells and its enhancement of cell killing by anti-neoplastic drugs, can play an important role in cancer chemotherapy.**

THE POSSIBILITY that hypoxic cells in solid tumours may limit successful local control of these tumours by X-rays has been recognized by radiation oncologists for more than a quarter of a century (Thomlinson & Gray, 1955). The realization that hypoxia may also be a factor of importance in chemotherapy is much more recent (Roizin-Towle & Hall, 1978).

Over the years, a substantial effort has been devoted to developing methods to overcome the problem of hypoxic cells in radiotherapy, the most recent of which involves the use of heterocyclic nitro compounds that mimic $O_2$ and interact with radiation to increase specifically the sensitivity of hypoxic cells, while not affecting the response of normal aerated cells (Adams & Cooke, 1969). A number of compounds have been identified and studied in experimental systems, and one of them, misonidazole (MISO), has already been introduced into clinical trials. In addition to preferentially sensitizing hypoxic cells, MISO is also preferentially cytotoxic to cells deficient in $O_2$. This has been reported for single cells *in vitro* (Hall & Roizin-Towle, 1975; Mohindra & Rauth, 1976; Moore *et al.*, 1976; Stratford & Adams, 1977), for cells in multicellular spheroids (Sutherland *et al.*, 1976) and in solid tumours in experimental animals (Denekamp & Harris, 1976; Brown, 1975; Fowler *et al.*, 1976). This cytotoxicity also proved to be strongly dependent on temperature (Stratford & Adams, 1977; Hall *et al.*, 1977). Hypoxia may limit the efficiency of chemotherapy in several ways (Roizin-Towle & Hall, 1978; Lin *et al.*, 1976). In some cases, drug availability may be a severe limitation, since cells deficient in $O_2$ are of necessity located some distance from a capillary. If $O_2$ does not reach them, some drugs may not either. A second factor is that cells at low $O_2$ tension are likely to be slow-cycling, or out of cycle altogether, making them relatively resistant particularly to drugs that are cycle-specific. Based on this reasoning, it was suggested that a compound such as MISO would be a logical addition to a chemotherapy cocktail to effectively kill the hypoxic cells that are resistant to conventional chemotherapy (Roizin-Towle & Hall, 1978).

This initial suggestion that the toxicity to hypoxic cells of electron-affinic agents might be valuable in combination with antineoplastic agents was based on the premise that they would act in a way that was complementary but independent, *i.e.*, that the hypoxia-mediated drugs would kill the hypoxic cells spared by the conventional chemotherapy agents. Sutherland *et al.* (1979) showed that when MISO was used to pretreat multicellular spheroids before the addition of Adriamycin, the number of clonogenic cells per spheroid was much less than with either agent alone, though the results are consistent with independent action of the two drugs.

There is now good evidence, however, of a direct interaction between electron-affinic compounds and other antineoplastic agents (Stratford *et al.*, 1980). Rose *et al.* (1980) investigated the response of the Lewis lung tumour in mice to the combined effects of MISO with antineoplastic agents, including Melphalan, cyclophosphamide, 5-fluorouracil and *cis*-platinum.

The present investigation extends the range of chemotherapy agents known to interact with MISO to include Bleomycin, and in particular addresses itself to the question of the mechanism of the interaction.

### MATERIALS AND METHODS

Chinese hamster V79 cells were used for these experiments, grown in GIBCO F10 culture medium, supplemented with 10% foetal calf serum and antibiotics.

Drug treatments were performed at 37·5°C, with the cells maintained in suspension in specially made glass vessels based on the design of Chapman & Urtasun (1977). These vessels include a magnetic stirrer to keep the cells in suspension, inlet and outlet portals to allow the suspension to be rendered aerated or hypoxic by the use of a suitable gas mixture, and a facility to allow cell samples to be withdrawn with a small pipette without significantly disturbing the $O_2$ status of the cell suspension.

On the day of an experiment, cells in exponential growth were harvested by trypsinization from several large Falcon culture flasks (75 $cm^2$) and prepared into a suspension which was shared between the various treatment vessels and diluted to a final concentration of $10^6$ cells/ml after the addition of the various drugs according to the plan of the particular experiment.

In many experiments to be described, cells were treated with MISO before exposure to various antineoplastic agents. In such cases, the cells were removed from suspension by centrifugation, so that the MISO could be diluted out before subsequent treatments with chemotherapy agents.

During treatment with MISO or various chemotherapy agents, cell samples were withdrawn from the cell suspension at frequent intervals, and various aliquots plated into culture flasks containnig fresh growth medium, to assay for colony formation. Care was taken to ensure that the drug concentration introduced into the flasks used to assess colony formation was not sufficient to affect the plating efficiency.

All drug solutions were made up fresh the day of an experiment in F10 culture medium. They were filter-sterilized and diluted with culture medium to the desired final concentration. Bleomycin was generously donated by Bristol Laboratories, Syracuse, New York. Melphalan and *cis*-platinum (*cis*-DDP) were supplied by the Drug Synthesis and Chemistry Branch of the National Cancer Institute. Cysteamine was purchased from Sigma Chemical Company of St Louis, Missouri.

At the conclusion of all drug treatments, the cell samples were plated into sealed Falcon flasks (25 $cm^2$) and incubated for 8 days at 37·5°C, when they were fixed, stained and the macroscopic colonies per flask were counted.

### RESULTS

The data presented in Figs 1–7 represent various sequences of drug combinations, involving MISO, cysteamine and 3 antineoplastic agents. Each experiment was repeated several times, with the same conclusions. However, in our experience with some antineoplastic agents *in vitro*, different drug batches vary so much that it is not practical to pool data from experiments performed over a period of time.

Consequently each figure includes data from one large self-contained experiment. Each point plotted is the mean of 3–6 replicate flasks and the error bars represent the standard error.

Fig. 1 shows the effect of pretreating cells under aerated or hypoxic conditions with or without 5mM MISO, before a further exposure in air to 100 $\mu$g/ml of Bleomycin for 0–3 h.

Fig. 1.—The left-hand panel shows the fraction of cells surviving the 3h pretreatment under air or hypoxia, with or without MISO. The right-hand panel shows the fraction of cells surviving treatment with BLM after the various pretreatments.

Fig. 2.—The left-hand panel shows the fraction of cells surviving the 3h pretreatment under hypoxia, with MISO, BLM, or no added drug. The right-hand panel shows the fraction of cells surviving treatment with BLM or MISO after various pre-treatments.

Fig. 3.—The left-hand panel shows the fraction of cells surviving a 3h pretreatment under hypoxia with or without 5mM MISO and 5mM cysteamine. The right-hand panel shows the fraction of cells surviving treatment with BLM after the various pretreatments.

Fig. 2 shows the results of an experiment designed to investigate the importance of the sequence of drug treatments. Cells were pretreated with MISO, followed by BLM, or *vice versa*; hypoxia was maintained throughout. It is evident that the fraction of cells surviving the two treatments is lower by two orders of magnitude when MISO precedes BLM, than with the reverse sequence.

The data in Fig. 3 show again that hypoxic pretreatment with MISO greatly enhances the subsequent cytotoxicity of BLM, but that the enhancement can be blocked to a large extent by the addition of an equimolar concentration of cysteamine during the pre-incubation.

Fig. 4 shows data from 2 experiments in which cells under aerated conditions received 0–5h exposure to 100 $\mu$g/ml of BLM, with or without simultaneous 5mM cysteamine. The data indicate that cysteamine decreases the effectiveness of BLM in cell killing when present at the same time.

Fig. 5 shows the effect of treating cells under hypoxic conditions with or without MISO, followed by an exposure in air to 0·5 $\mu$g/ml of Melphalan. The data in this figure show first, that pretreatment with MISO enhances subsequent cell killing by Melphalan, and second, that this enhancement can be largely reversed if the pre-

FIG. 4.—Data to show the cytoxicity of BLM at 100 $\mu$g/ml for various periods of time under aerated conditions (solid symbols) and the extent to which this cytotoxicity can be blocked by the simultaneous administration of 5mM cysteamine (open symbols).

FIG. 5.—The left-hand panel shows the fraction of cells surviving a 3h pretreatment under hypoxia with or without MISO and/ or cysteamine. The right-hand panel shows the fraction of cells surviving treatment with Melphalan (0·5$\mu$ g/ml) after the various pretreatments.

FIG. 6.—The left-hand panel shows the fraction of cells surviving a 3h hypoxic pretreatment with or without MISO and/or cysteamine. Also shown is the 3 h survival for cells exposed in air to 15$\mu$m *cis*-DDP. The right-hand panel shows the fraction of cells surviving various treatments of *cis*-DDP at 15$\mu$M after the various pretreatments, or of cells surviving treatment with MISO under hypoxia, after 15$\mu$M *cis*-DDP in air.

treatment with MISO involves a simultaneous exposure to 5mM cysteamine.

The data in Fig. 6 show the effect of pretreating cells with MISO under hypoxic conditions, with or without cysteamine, and the subsequent response in air to 15$\mu$M *cis*-DDP. The enhancement by MISO of *cis*-DDP toxicity is shown, and also that treating cells with *cis*-DDP *before* MISO has no apparent enhancing effect. The data also show that the presence of cysteamine during the pretreatment with MISO reduces the enhancement of the *cis*-DDP cytotoxicity.

### DISCUSSION

The experiments described confirm previous reports in the literature that pretreatment with MISO strikingly potentiates the cytotoxicity of Melphalan and *cis*-DDP (Stratford *et al.*, 1980) while adding the new information that BLM killing is also greatly enhanced. The potentiation produced by pretreatment with MISO occurs only if this pretreatment is under hypoxia. The subsequent exposure to the chemotherapeutic agent may be under aerated or hypoxic conditions; this has been shown for BLM in the present paper, but other unpublished data indicates that it is true also for Melphalan and *cis*-DDP.

The initial idea that MISO may have a place in a chemotherapy cocktail, to sterilize the hypoxic cells that are resistant to killing by the more conventional antineoplastic agents (Roizin-Towle & Hall, 1978) now gives way to the wider claim that there is an interaction between the two classes of agents, *i.e.* that MISO potentiates a wide range of chemotherapeutic agents.

The present experiments, designed to reveal the mechanism of these effects, demonstrate that the concomitant application of cysteamine during the pretreatment with MISO inhibits the enhancement by MISO of the subsequent cytotoxicity of BLM, *cis*-DDP and Melphalan. Since cysteamine is a free-radical scavenger, free radicals are strongly implicated in the mechanism of the enhancement of the action of chemotherapeutic agents by MISO (Hall & Biaglow, 1977). The inhibition by cysteamine of the cytotoxicity of BLM (Fig. 4) is strong circumstantial evidence that the action of this drug is also mediated through free radicals, which correlates with previous findings (Buettner & Oberley, 1979; Fukuoka *et al.*, 1980).

MISO has now been shown to have three distinct properties:

*First*, it preferentially sensitizes hypoxic cells to killing by X-rays. The enhancement of radiosensitivity is dose-modifying if the radiation is delivered shortly after the addition of MISO, but if cells are pre-incubated under hypoxia for some hours before irradiation, the radiosensitization is increased, and MISO ceases to be dose-modifying, in that the shoulder to the survival curve is reduced (Hall & Biaglow, 1977; Wong *et al.*, 1978).

*Second*, it is cytotoxic to mammalian cells, though extended contact for several hours is necessary for substantial cell killing to become apparent. Again hypoxic cells are affected differentially since 100-fold concentrations are required for equal cell killing in aerated cells.

*Third*, pre-incubation with MISO potentiates or enhances the cytotoxicity produced by a subsequent exposure to a number of chemotherapeutic agents, including alkylating agents and antibiotics. In experiments with cells cultured *in vivo*, the pretreatment must be under hypoxia for the maximum enhancement of cytotoxic effect, but in solid tumours in laboratory animals, the degree of potentiation is perhaps too much to be explained as an effect on hypoxic cells only.

The mechanism for hypoxic-cell toxicity by MISO is believed to be the metabolic reduction of the nitro group to stable intermediates, which then bind to cellular macromolecules causing DNA damage and interfere with electron transport (Olive, 1980). While depletion of thiols plays an important role in hypoxia-mediated cytotoxicity by MISO, other biochemical mechanisms must clearly play a role as well (Kinnula & Hassinen, 1980). These other effects were demonstrated at the ultrastructural level, where treating cells under hypoxia with MISO for 3 h produced such severe damage to the endoplasmic reticulum and mitochondria that damage to glycolytic processes and malfunctioning enzymic repair processes seemed inescapable (Roizin-Towle *et al.*, 1977). It appears that many factors are operating, and a complicated interplay of biochemical reactions may occur during hypoxia (Rudolf, 1980)—an area of research that requires further investigation, especially with regards to cancer-drug metabolism, toxicity and interaction with other agents.

At the mechanistic level, the question is whether these diverse properties of MISO are related. The straightforward radiosensitizing properties are adequately accounted for in terms of the electron affinity of MISO and in its ability to mimic

$O_2$. The other properties, including the changing radiosensitization with time, the cytotoxicity of MISO and its ability to potentiate the cytotoxicity of various chemotherapeutic agents, all require prolonged incubation of cells with the drug under hypoxic conditions. This in turn involves the breakdown and metabolism of the drug (Whitmore & Guylas, 1980). It is possible, therefore, that a common mechanism may be involved.

In the report which first described the increase of radiosensitization with storage (Hall & Biaglow, 1977) it was postulated that this was due to the depletion in the cells of natural sulphydryl compounds, particularly thiols. The substantial body of new data, as well as the new phenomena discovered since, appear to be compatible with this hypothesis. In particular, the potentiation of chemotherapy agents by pretreatment with MISO is readily explained in these terms. Prolonged exposure to MISO under hypoxia is required to deplete the cells of thiols, following which the cells are very sensitive to killing by chemotherapy agents—particularly those that produce their cytotoxicity *via* free radicals. The replacement of these thiols by cysteamine, as shown in these data, lends support to this idea. Free radicals are also involved in the cytotoxicity of BLM, which can be blocked by the addition of cysteamine, a radical scavenger. On the other hand, the cytotoxicity of BLM is greatly enhanced when cells are pretreated with MISO.

This effect of MISO is likewise remembered by cells when they are subsequently treated with Melphalan or *cis*-DDP, showing that it enhances the action of other chemotherapeutic agents as well. Since MISO shows little toxicity to aerated cells, its use in combination with other established cancer drugs would be beneficial for two reasons. First, it would selectively kill hypoxic cells and secondly, it would subsequently reduce the systemic dose of chemotherapeutic drugs needed to produce a certain level of cell kill not achievable on their own.

The investigation described here lends strong support to the contention that the hypoxia-mediated electron-affinic compounds such as Misonidazole, developed initially as radiosensitizers, have a place in chemotherapy. Their selectivity for hypoxic cells, and their enhancement of killing by chemotherapeutic agents, show the importance for a calculated exploitation of drug-delivery sequences.

This investigation was supported by Grant No. CA 18506 to the Radiological Research Laboratory/ Department of Radiology and Grant No. CA 13696 to the Cancer Center/Institute of Cancer Research awarded by the National Cancer Institute, DHHS.

### REFERENCES

Adams, G. E. & Cooke, M. S. (1969) Electron affinic sensitization. I. A structural basis for clinical radiosensitizers in bacteria. *Int. J. Radiat. Biol.*, **15**, 457.

Brown, J. M. (1975) Selective radiosensitization of hypoxic cells of mouse tumors with Metronidazole and Ro-07-0582. *Radiat. Res.*, **62**, 633.

Buettner, G. R. & Oberley, L. W. (1979) The production of hydroxyl radical by Tallysomycin and copper (II). *FEBS Lett.*, **101**, 333.

Chapman, J. D. & Urtasun, R. C. (1977) The application in radiotherapy of substances which modify cellular radiation response. *Cancer*, **40**, 484.

Denekamp, J. & Harris, S. R. (1976) The response of transplantable tumor to fractionated irradiation. I. X-rays and the hypoxic cell radiosensitizer Ro-07-0582. *Radiat. Res.*, **66**, 66.

Fowler, J. F., Adams, G. E. & Denekamp, J. (1976) Radiosensitizers of hypoxic cells in solid tumors. *Cancer Treatment Rev.*, **3**, 227.

Fukuoka, T., Muraoka, Y., Fujii, A., Naganawa, H., Takita, T. & Umezawa, H. (1980) Chemistry of Bleomycin. XXV. Reductive methylation of Bleomycin: A chemical proof for the presence of the free secondary amine in Bleomycin. *J. Antibiots (Tokyo)*, **33**, 114.

Hall, E. J., Astor, M., Geard, C. & Biaglow, J. (1977) Cytotoxicity by Ro-07-0582: Enhancement by hyperthermia and protection by cysteamine. *Br. J. Cancer*, **35**, 809.

Hall, E. J. & Biaglow, J. (1977) Ro-07-0582 as a radiosensitizer and cytotoxic agent. *Int. J. Radiat. Oncol. Biol. Phys.*, **3**, 521.

Hall, E. J. & Roizin-Towle, L. A. (1975) Hypoxic sensitizers: Radiobiological studies at the cellular level. *Radiology*, **117**, 453.

Kinnula, V. L. & Hassinen, I. (1978) Metabolic adaption to hypoxia. *Acta Physiol. Scand.*, **104**, 109.

Lin, A. J., Cosby, L. A. & Sartorelli, A. C. (1976) Potential bioreductive alkylating agents. *ACS Symposium Series, No. 30, Cancer Chemotherapy*, p. 71.

Mohindra, J. K. & Rauth, A. M. (1976) Increased cell killing by metronidazole and nitrofurazone of hypoxic compared to aerobic mammalian cells. *Cancer Res.*, **36**, 930.

MOORE, B. A., PALCIC, B. & SKARGARD, L. D. (1976) Radiosensitization and toxic effects of the 2-nitroimidazole Ro-07-0582 in hypoxic mammalian cells. *Radiat. Res.*, **67**, 459.

OLIVE, P. L. (1980) Mechanisms of the *in vitro* toxicity of nitroheterocyclics, including Flagyl and Misonidazole. In *Radiation Sensitizers: Their Use in the Clinical Management of Cancer* (Ed. Brady). USA: Masson Publ. p. 39.

ROIZIN-TOWLE, L. & HALL, E. J. (1978) Studies with Bleomycin and Misonidazole on aerated and hypoxic cells. *Br. J. Cancer*, **37**, 254.

ROIZIN-TOWLE, L., ROIZIN, L., HALL, E. J. & LIU, J. C. (1977) Effects of Misonidazole on the ultrastructure of mammalian cells cultured *in vitro*. *Radiat. Res.*, **74**, 471.

ROSE, C. M., MILLAR, J. L., PEACOCK, J. H., PHELPS, T. A. & STEPHENS, T. C. (1980) Differential enhancement of Melphalan cytotoxicity in tumor and normal tissue by Misonidazole. In *Radiation Sensitizers: Their Use in the Clinical Management of Cancer* (Ed. Brady). USA: Masson Publ. p. 250.

RUDOLF, M. (1978) Hypoxia: Its patho-physiology. *Br. J. Hosp. Med.*, **400**, 35.

STRATFORD, J. I. & ADAMS, G. E. (1977) Effect of hyperthermia on the differential cytotoxicity of the hypoxic cell radiosensitizer, the 2-nitroimidazole Ro-07-0582, on mammalian cells *in vitro*. *Br. J. Cancer*, **35**, 307.

STRATFORD, I. J., ADAMS, G. E., HORSMAN, M. R. & 4 others (1980) The interaction of Misonidazole with radiation, chemotherapeutic agents, or heat: A preliminary report. In *Radiation Sensitizers: Their Use in the Clinical Management of Cancer* (Ed. Brady). USA: Masson Publ. p. 276.

SUTHERLAND, R. M., EDDY, H. A., BAREHAM, B., REICH, K. & VANANTWERP, D. (1979) Resistance to Adriamycin in multicellular spheroids. *Int. J. Radiat. Oncol. Biol. Phys.*, **5**, 1225.

SUTHERLAND, R. M., KOCH, C. J., BIAGLOW, J. E. & SRIDHAR, R. (1976) Potential chemotherapeutic drugs with selective toxicity for hypoxic cells. *Am. Assoc. Cancer Res.*, **17**, 60.

THOMLINSON, R. H. & GRAY, L. H. (1955) The histologic structure of some human lung cancers and the possible implications for radiotherapy. *Br. J. Cancer*, **9**, 539.

WHITMORE, G. F. & GUYLAS, S. (1980) Lethal and sublethal effects of Misonidazole under hypoxic conditions. In *Radiation Sensitizers: Their Use in the Clinical Management of Cancer* (Ed. Brady). USA: Masson Publ. p. 99.

WONG, T. W., WHITMORE, G. F. & GUYLAS, S. (1978) Studies on the toxicity and radiosensitizing ability of Misonidazole under conditions of prolonged incubation. *Radiat. Res.*, **75**, 541.